# Prevalence and Predictors of Additional Ablation Beyond Pulmonary Vein Isolation in Patients With Paroxysmal Atrial Fibrillation

**DOI:** 10.3389/fcvm.2021.690297

**Published:** 2021-07-20

**Authors:** Xin Xie, Gang Yang, Xiaorong Li, Jinbo Yu, Fengxiang Zhang, Weizhu Ju, Hongwu Chen, Mingfang Li, Kai Gu, Dian Cheng, Xuecheng Wang, Yizhang Wu, Jian Zhou, Xiaoqian Zhou, Baowei Zhang, Pipin Kojodjojo, Kejiang Cao, Bing Yang, Minglong Chen

**Affiliations:** ^1^Department of Cardiology, Shanghai East Hospital, School of Medicine, Tongji University, Shanghai, China; ^2^Department of Cardiology, The First Affiliated Hospital of Nanjing Medical University, Nanjing, China; ^3^Division of Cardiology, Ng Teng Fong General Hospital, Singapore, Singapore

**Keywords:** atrial fibrillation, catheter ablation, additional ablation, concomitant arrhythmia, non-pulmonary vein trigger

## Abstract

**Background:** Pulmonary vein isolation (PVI) is an effective strategy in the treatment of paroxysmal atrial fibrillation (PAF). Yet, there are limited data on additional ablation beyond PVI. In this study, we sought to assess the prevalence, predictors, and outcomes of additional ablation in PAF patients.

**Methods:** A total of 537 consecutive patients with PAF were retrospectively evaluated for the index procedure. PVI was successfully conducted in all patients, after which electrophysiological study and drug provocation were performed, and additional ablations were delivered for concomitant arrhythmias, non-PV triggers, and low voltage zone (LVZ). The prevalence, predictors, and outcomes of additional ablation were analyzed.

**Results:** Among 537 consecutive patients, 372 addition ablations were performed in 241 (44.88%) patients, including 252 (67.74%) concomitant arrhythmias in 198 (36.87%) patients, 56 (15.05%) non-PV triggers in 52 (9.68%) patients and 64 (17.20%) LVZ modification in 47 (8.75%) patients. Lower LVEF (OR = 0.937, *p* = 0.015), AF episode before procedure (OR = 2.990, *p* = 0.001), AF episode during procedure (OR = 1.998, *p* = 0.002) and AF episode induced after PVI (OR = 15.958, *p* < 0.001) were independent predictors of additional ablation. Single-procedure free from atrial arrhythmias at 58.36 ± 7.12 months post-ablation was 70.48%.

**Conclusions:** Additional ablations were common in patients with PAF for index procedure. Lower LVEF and AF episodes before, during the procedure, and induced after PVI predicts additional ablation.

## Introduction

Since Haissaguerre et al. identified pulmonary veins (PVs) foci as major triggers of atrial fibrillation (AF) (1), pulmonary vein isolation (PVI) has been widely accepted as the basis of AF ablation procedures (2). Nowadays, PVI in paroxysmal atrial fibrillation (PAF) patients has reached a success rate of 46–56% (3–5) after long follow-up.

However, the PV foci are not always the only target, and PVI alone might not guarantee a long-term AF-free outcome in PAF patients. Considering AF is a progressive and complex type of arrhythmia that involves atrial substrate remodeling (2), various strategies have been tried beyond PVI, including linear ablation, complex fractionated atrial electrogram (CFAE) guided ablation, ganglionated plexus modification and focal impulse and rotor modulation (FIRM) targeting (6–9). Meanwhile, non-PV triggers and concomitant supraventricular arrhythmias also account for a large portion of ablation beyond PVI (10–12). In our center, addition ablations were mainly performed due to concomitant arrhythmias, non-PV triggers, and limited substrate modifications in PAF patients who underwent index procedure. Herein, we reported our single-center experience on the prevalence, characteristics, predictors, and outcomes of additional ablation in such population.

## Methods

### Study Population

**Figure 1:**
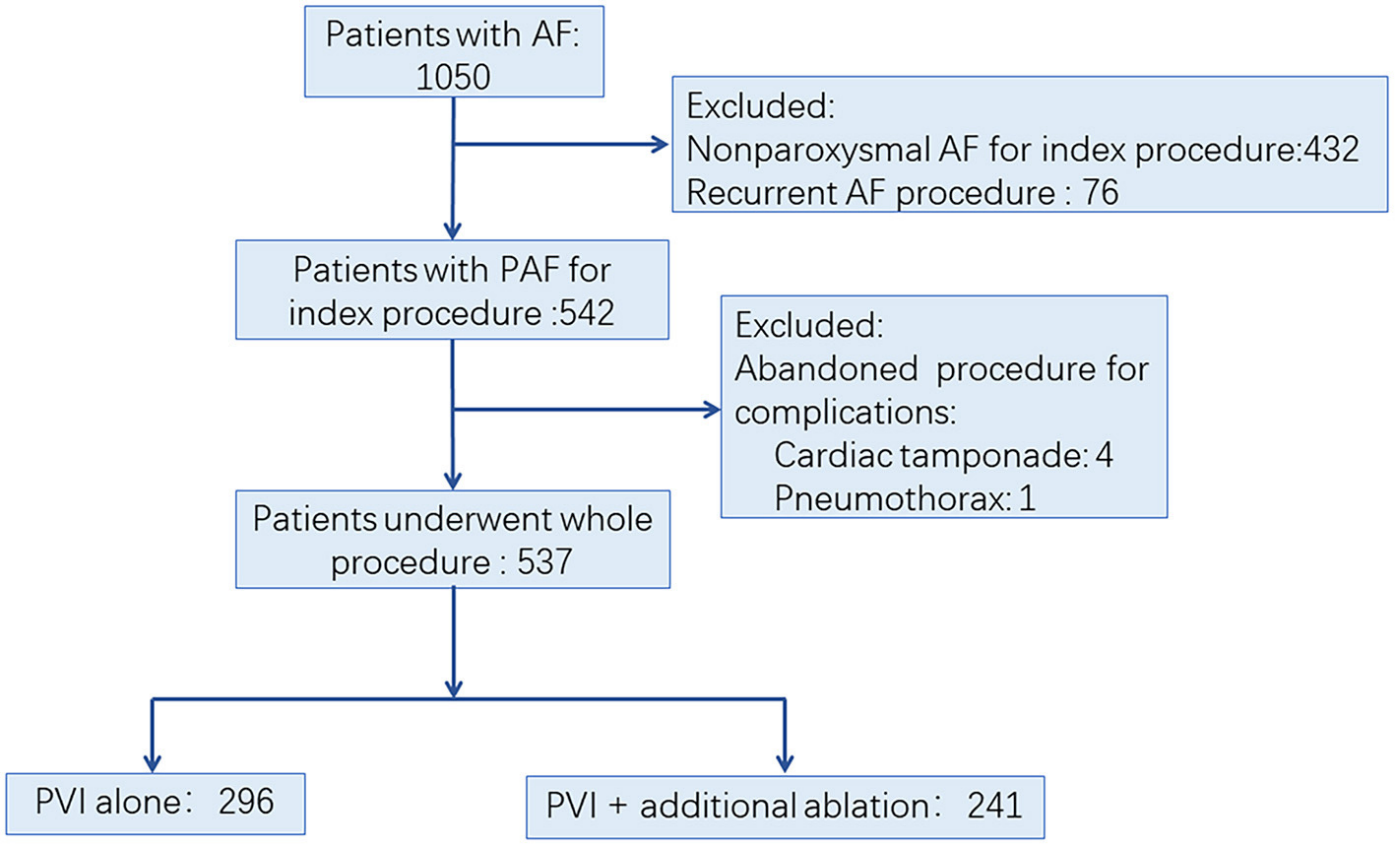
Trial profile. AF, atrial fibrillation; PAF, paroxysmal atrial fibrillation; PVI, pulmonary vein isolation.

We retrospectively evaluated consecutive patients with AF in the First Affiliated Hospital of Nanjing Medical University between Jan 1, 2014, and Dec 31, 2015. The AF ablation volume in our center is more than 600 per year. The procedures were performed by physician with an experience of more than 100 cases per year. PAF was defined as AF that terminated spontaneously or with intervention within 7 days of onset (2). The inclusion criteria included: (1) patients aged 18 to 80 years; (2) patients diagnosed with PAF; (3) no left atrium (LA) thrombosis was detected before the procedure. The exclusion criteria were the following: (1) patients with non-paroxysmal AF; (2) patients who underwent AF ablation before; (3) abandoned procedure due to occurrence of complications. The process of patient enrollment is shown in Figure 1. The study was approved by the Institutional Review Board of Nanjing Medical University. All data were collected from hospital medical record system and stored by a specially-assigned person. All patient identifier were removed before statistical analysis.

### PVI Procedure

All patients were prescribed oral anticoagulants for at least 3 weeks. Antiarrhythmic agents except amiodarone were discontinued for at least 5 half-lives. Informed consent was obtained, and LA thrombus was excluded using transesophageal echocardiography before the procedure. Procedures were performed under local anesthesia and intravenous fentanyl. Access was obtained *via* bilateral femoral veins. A decapolar catheter was advanced into the coronary sinus and a quadripolar catheter was placed at the His bundle region. Double transseptal access was performed using 8.5F sheaths (SL1, St. Jude Medical, MN, USA). Intravenous heparin was given to maintain an activated clotting time of 300 ± 50 s.

LA was reconstructed using electroanatomic mapping systems (CARTO, Biosense Webster, CA, USA or EnSite-NavX, St Jude Medical, MN, USA). PV orifices were identified by selective venography and catheter drop movement. Antral circumferential ablation was carried out around pairs of ipsilateral PVs using an open-irrigation ablation catheter (Thermocool for CARTO; CoolFlex for ExSite NavX). A power limit of 35 W, a tip temperature limit of 43°C, and an infusion rate of 17 mL/min was adopted. Moreover, power of 30 W was selected when ablating on the posterior wall. PVI was defined as the abolition or dissociation of PV potentials with the circular mapping catheter. AF persisting after PVI was terminated by direct current cardioversion (DCCV).

### Definition and Categories of Perioperative AF Episodes

Perioperative AF episodes were defined as any AF episode lasting >30 s during the perioperative period. The subtype of perioperative AF episode was categorized as: (1) AF episode before the procedure was defined as AF that presented before venous puncture. (2) AF episode during the procedure was defined as AF episode that presented from the venous puncture to PVI. (3) AF episode needing DCCV was defined as AF sustained after PVI and needing DCCV to conversion. (4) AF episode induced after PVI was defined as AF induced by electrophysiological study and/or drug provocation and sustained more than 5 min after PVI.

### Additional Ablation for Concomitant Arrhythmia, Non-PV Triggers and Substrate Modification

Concomitant arrhythmia was defined as any pre-procedural documented, spontaneous or induced sustained atrial tachyarrhythmia, supraventricular tachycardia (SVT) or symptomatic premature atrial contraction (PAC) except for AF during the procedure. The non-PV trigger was defined as recurrent PAC that originated outside the PVs and initiates AF (13). All concomitant arrhythmias and non-PV triggers were mapped and ablated (11, 14, 15). Substrate mapping and modification targeting the low voltage zone (LVZ) have been described in our previous study (16). In brief, high-density mapping was performed after PVI during sinus rhythm or high right atrium pacing. Areas with low-voltage (<0.4 mV) and abnormal local intracardiac electrograms (multiphasic electrogram with ≥3 positive or negative distinct peaks and electrogram duration ≥50 ms) were targeted for further ablation.

### Electrophysiological Study and Drug Provocation After PVI

**Figure 2:**
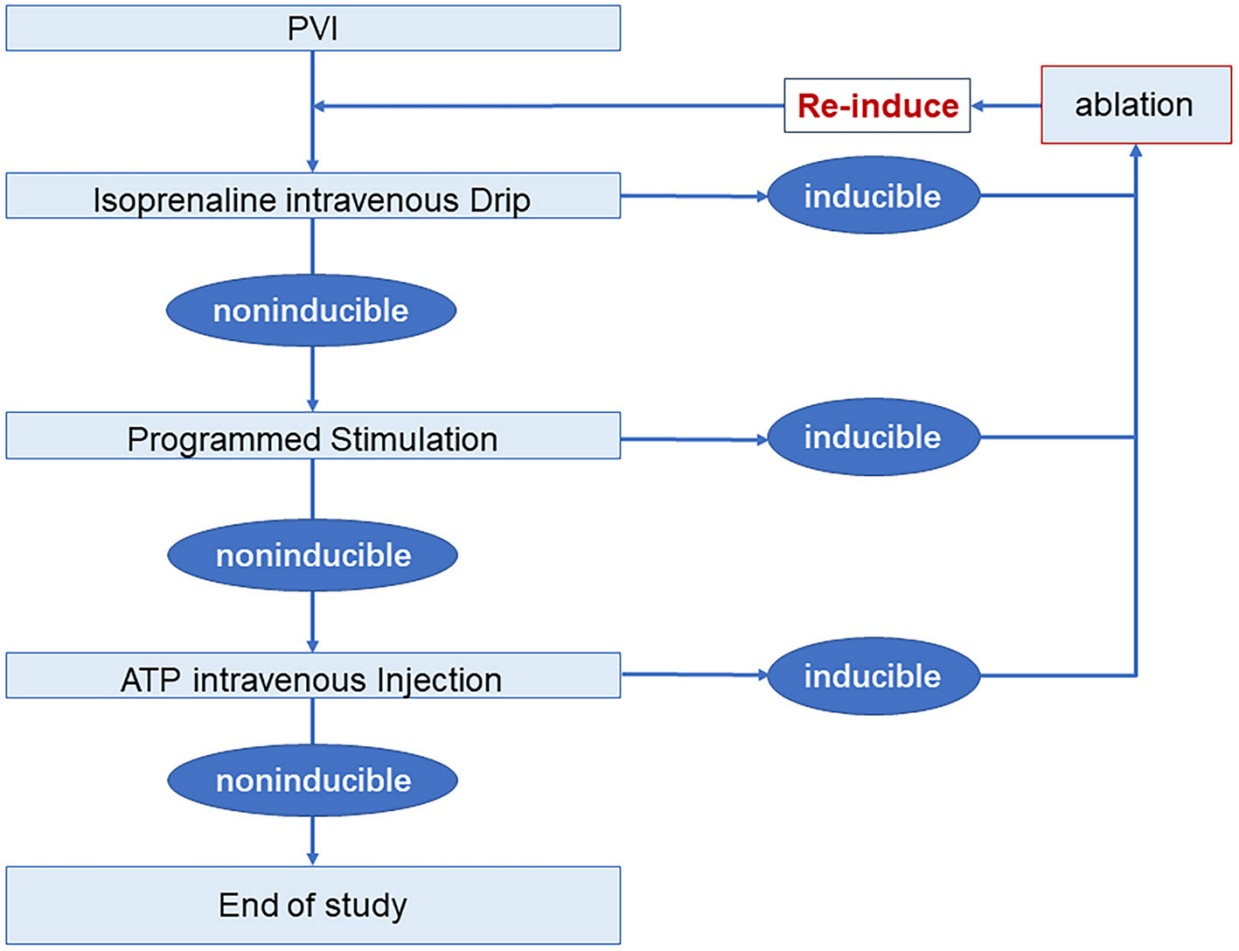
Electrophysiology study and drug provocation protocol. AF, atrial fibrillation; ATP, adenosine triphosphate; PVI, pulmonary vein isolation.

Electrophysiological study and drug provocation after PVI were performed in all patients. A 30-min observation period was used to assess spontaneous recovery of PV connection, during which, isoproterenol was intravenously given 4–20 ug/min to achieve a 20% increase of baseline heart rate. Programmed stimulation was given with 3 basal baseline cycle lengths (500 ms, 400 ms, and 330 ms) and up to 3 extra stimulations at either the high right atrium or coronary sinus ostium. A 40 mg of adenosine triphosphate (ATP) was used as an intravenous bolus to evaluate dormant conduction or non-PV trigger. PV reconnection, concomitant arrhythmias, and non-PV trigger were mapped and ablated. Electrophysiological study and drug provocation were re-performed until these could no longer be elicited (Figure 2). If only AF was induced, no additional ablations were delivered. And, if AF persisted, DCCV was performed to restore sinus rhythm.

### Follow-Up

Oral anticoagulation therapy and antiarrhythmic drugs were prescribed for all patients for at least 2–3 months after the procedure. During the first year, all patients were followed-up through clinic visits and 24-h Holter recordings at 1, 3 (the blanking period), 6, and 12 months. In subsequent years, telephone interviews, clinic visits, and 24-h Holter recordings were undertaken every 6 months. A pulse measurement and ECG recording were recommended whenever patients were symptomatic. Successful ablation was defined as no atrial tachyarrhythmias lasting more than 30 s after the blanking period, without antiarrhythmic drugs (2).

### Statistical Analysis

The continuous variables are expressed as the mean ± SD. Categorical variables are expressed as number and percentage. The continuous variables were analyzed with an unpaired *t*-test or Wilcoxon analysis. Categorical variables were compared with the Chi-square test or Fisher's exact test. The event-free rates were calculated using Kaplan–Meier analysis, while log-rank statistics were used for group comparisons. The outcome is unknown for patients who did not reach the event during follow-up due to loss to follow-up or dying. In such cases, the time of follow up was recorded and interpreted as censored data. Univariate and multivariable logistic regression analyses and Cox regression were performed to assess independent predictors associated with additional ablation. The results are expressed as *p* values. Factors with *p* < 0.15 in univariate analyses were enrolled in multivariate analyses. A *p* value of <0.05 was considered as statistically significant. All statistical analyses were performed using SPSS software version 20.0.

## Results

### Patient Characteristics

Among 1,050 consecutive patients who underwent AF ablation between Jan 1, 2014, and Dec 31, 2015, a total of 513 patients were excluded due to non-paroxysmal AF ablation (*n* = 432), non-index procedure (*n* = 76), and abandoned procedure (*n* = 5), respectively. Finally, 537 PAF patients were enrolled in this study (Figure 1). Among them, 296 (55.12%) patients underwent PVI alone (Group I), while 241 (44.88%) patients had additional ablation beyond PVI (Group II). Detailed baseline characteristics of these patients are shown in Table 1. Compared with Group I, there was significantly higher CHA2DS2-VASc score, larger LA diameter, and lower left ventricular ejection fraction (LVEF) in patients in Group II, respectively (Table 1).

**Table 1:** Baseline characteristics.

**Patient characteristics**	**Total (*n* = 537)**	**Group I (*n* = 296)**	**Group II (*n* = 241)**	***P* Value**
Age (years)	58.64 ± 1.40	58.25 ± 9.76	59.12 ± 11.13	0.340
Male (%)	328 (61.08%)	189 (63.85%)	139 (57.68%)	0.144
History of PA (Month)	38.44 ± 55.23	36.39 ± 51.29	40.81 ± 59.68	0.364
Smoke (%)	88 (16.39%)	53 (17.91%)	35 (14.52%)	0.292
Alcohol consumption (%)	70 (13.04%)	38 (12.84%)	32 (13.28%)	0.880
Coronary artery disease (%)	50 (9.31%)	31 (10.47%)	19 (7.88%)	0.304
Congestive heart failure (%)	3 (0.56%)	0 (0%)	3 (1.24%)	0.090
Hypertension (%)	253 (47.11%)	145 (48.99%)	108 (44.81%)	0.335
Diabetes mellitus (%)	57 (10.61%)	28 (9.46%)	29 (12.03%)	0.336
CHA2DS2-VASc (%)				
0	206 (38.36%)	111 (37.50%)	95 (39.42%)	0.031
1	158 (29.42%)	101 (34.12%)	57 (23.65%)	
2	97 (18.06%)	50 (16.89%)	47 (19.50%)	
≥3	76 (14.15%)	34 (11.49%)	42 (17.43%)	
Echocardiography				
LA (mm)	36.45 ± 4.83	35.99 ± 4.36	37.01 ± 5.32	0.019
LVEF (%)	64.30 ± 3.92	64.70 ± 2.64	63.77 ± 4.98	0.011

*LAD, diameter of left atrium; LVEF, left ventricular ejection fraction; PAF, paroxysmal atrial fibrillation*.

### Perioperative AF Episodes Patterns

AF episodes before the procedure, AF episodes during the procedure, AF episodes needing DCCV and AF episode induced after PVI were documented in 54 (10.06%), 110 (20.48%), 47 (8.75%) patients, and 26 (4.84%) patients, respectively (Table 2). Compared with Group I, the patients in Group II showed a higher prevalence of AF episodes before the procedure (15.35 vs. 5.74%, *p* < 0.001), AF episodes during the procedure (25.31 vs. 16.55%, *p* = 0.001), AF episodes needing DCCV (14.11 vs. 4.39%, *p* < 0.001), and AF episode induced after PVI (9.96 vs. 0.68%, *p* < 0.001), respectively.

**Table 2:** AF episodes pattern.

**AF episodes pattern**	**Total (*n* = 537)**	**Group I (*n* = 296)**	**Group II (*n* = 241)**	***P* value**
AF episode before the procedure	54 (10.06%)	17 (5.74%)^‡^	37 (15.35%)^‡^	<0.001
AF episode during the procedure	110 (20.48%)	49 (16.55%)^†^	61 (25.31%)^†^	0.001
AF episode needing DCCV	47 (8.75%)	13 (4.39%)^‡^	34 (14.11%)^‡^	<0.001
AF episode induced after PVI	26 (4.84%)	2 (0.68%)^‡^	24 (9.96%)^‡^	<0.001

*AF, atrial fibrillation; DCCV, direct current cardioversion; PVI, pulmonary vein isolation*.

† *p <0.05*.

‡ *p <0.001*.

### PVI and Additional Ablations

**Figure 3:**
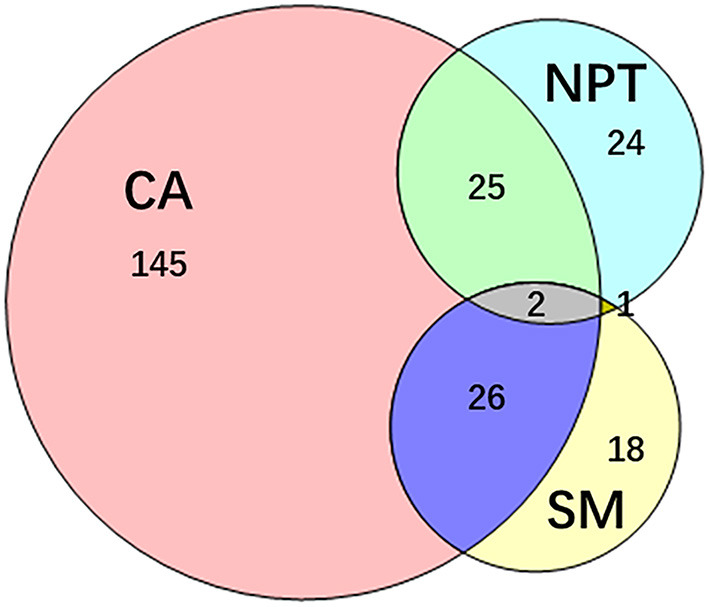
Additional ablation after PVI. 241 patients underwent additional ablation after PVI. The additional ablation mainly contains the ablation of concomitant arrhythmias, non-PV trigger elimination, substrate modification and their combinations. CA, concomitant arrhythmia; NPT, non-PV trigger; PVI, pulmonary vein isolation; SM, substrate modification.

PVI was achieved in all patients. Totally 372 additional ablations were performed in 241 (44.88%) patients. Among them, 145 (27%) patients presented 252 (67.74%) concomitant arrhythmias alone, 24 (4.47%) patients presented non-PV trigger alone, and 18 patients (3.35%) only underwent substrate modifications. Combinations of different categories of additional ablation was performed in 54 (10.6%) patients (Figure 3).

### Characteristics of Concomitant Arrhythmias

Concomitant arrhythmias, which accounted for the majority of additional ablations, were found in 198 (82.16%) patients. In terms of concomitant arrhythmia type, the most common was atrial flutter (AFL, 55.16%), followed by atrial tachycardia (AT, 24.60%), SVT (13.89%), and non-PV PACs (6.35%), respectively. Concomitant arrhythmias were mostly diagnosed according to previously documented ECG (37.30%) or perioperative episode (30.16%). During PVI, spontaneously AF converted to AFL, and AT was presented in nine and one patient, respectively. Concomitant arrhythmia was induced by electrophysiological study and drug provocation in 51 (20.24%) patients, wherein programmed stimulation, isoproterenol, and ATP accounted for 35 (13.89%), 10 (3.97%), and 6 (2.38%) patients, respectively. Subjective cavotricuspid isthmus linear ablation was performed in 16 patients with the suspicion of the diagnosis of AFL. Previously successfully ablated AFL and SVT were documented in four and one patient, respectively (Table 3).

**Table 3:** Concomitant arrhythmias in the index procedure of PAF.

**Concomitant Arrhythmia**	***N***	**Preoperative documented (*n*)**	**Spontaneous onset (*n*)**	**Provocation with Iso (*n*)**	**Programmed stimulation (*n*)**	**Provocation with ATP (*n*)**	**Conversion from AF (*n*)**	**Subjective ablation (*n*)**	**Previous ablation (*n*)**
AFL	139 (55.16%)	82	18	3	6	1	9	16	4
AT	62 (24.60%)	2	39	5	11	4	1	0	0
SVT	35 (13.89%)	9	7	0	18	0	0	0	1
Non-PV PACs	16 (6.35%)	1	12	2	0	1	0	0	0
Total	252 (100%)	94 (37.30%)	76 (30.16%)	10 (3.97%)	35 (13.89%)	6 (2.38%)	10 (3.97%)	16 (6.35%)	5 (1.98%)

*AF, atrial fibrillation; AFL, atrial flutter; AT, atrial tachycardia; ATP, adenosine triphosphate; PAC, premature atrial contraction; PAF, paroxysmal atrial fibrillation; PV, pulmonary vein; Iso, isoprenaline; SVT, supraventricular tachycardia*.

### Characteristics of Non-PV Trigger

Fifty-six non-PV triggers were documented in 52 patients (9.68%). Among them, four patients presented more than one origin of non-PV triggers. Non-PV triggers were spontaneous onset that was found in 33 (58.93%) patients, and those induced by electrophysiological study and drug provocation in 19 (33.93%) patients. Arbitrary superior vena cava (SVC) isolation was conducted in 4 (7.14%) patients, according to the operator's opinion. Non-PV triggers mostly originated from SVC and the detailed distribution were listed in Supplemental Table 1.

### Characteristics of LVZ Modification

A total of 64 LVZ modifications were performed in 47 patients. Twenty-one (32.81%) LVZ modifications were applied at LA anterior wall, followed by 9 (14.06%) at LA posterior wall, 9 (14.06%) at LA roof, 6(9.38%) at the right atrium, 6 (9.38%) at septal, 6 (9.38%) at right PV antrum, 3 (4.69%) at left atrial appendage, 3 (4.69%) at mitral valve isthmus, 1 (1.56%) at Marshall ligament, respectively. Sole LVZ was located in 33 (70.21%) patients, while 2 and 3 LVZs were noted in 10 (21.28%) and 4 (8.51%) patients, respectively.

### Predictors of Additional Ablation

Baseline variables and AF episodes patterns were fitted to univariate logistic regression analysis for assessing the predictors of additional ablation. Multi-variable logistic regression analysis revealed that lower LVEF (OR = 0.937, *p* = 0.015), AF episode before the procedure (OR = 2.990, *p* = 0.001), AF episode during the procedure (OR = 1.998, *p* = 0.002) and AF episode induced after PVI (OR = 15.958, *p* < 0.001) were independent predictors for additional ablation (Supplemental Table 2).

### Follow-Up and Predictors for Recurrence

**Figure 4:**
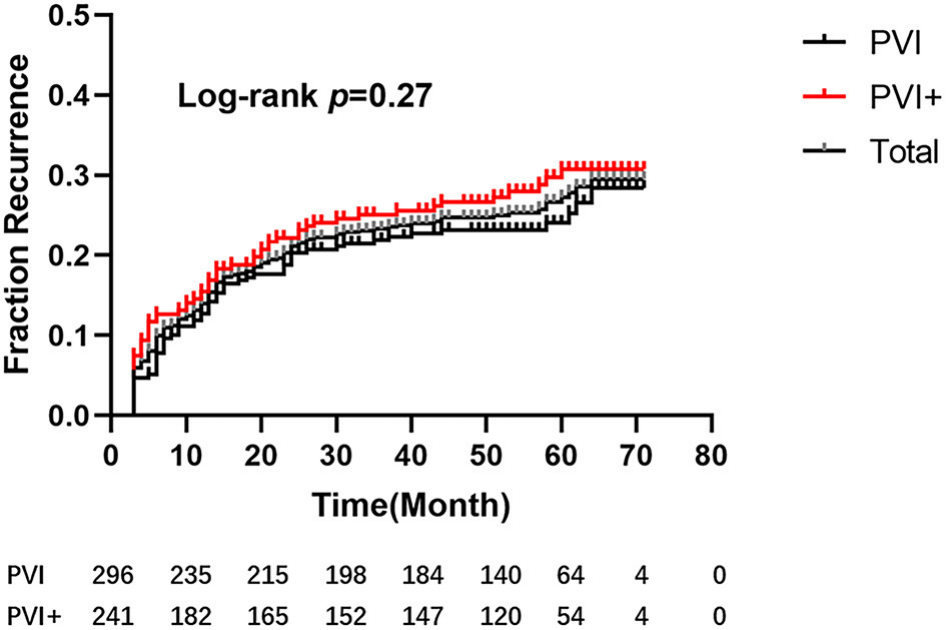
Kaplan–Meier estimation of freedom from atrial tachyarrhythmia after a single procedure. After a mean follow-up period of 58.36 ± 7.12 months, there were no significant differences between the 2 groups (hazard ratio [HR], 0.82; 95% confidence interval [CI], 0.58–1.16; *P* = 0.27).

After a mean follow-up period of 58.36 ± 7.12 months, five patients (one in Group I, the other four in Group II) died: three due to respiratory diseases, 1 due to stroke, and 1 due to unknown reason. Ninety-five patients (56 in Group I, 39 in Group II) were lost to follow-up. One hundred and twenty-nine patients, 66 in Group I and 63 in Group II, recurred. The single-procedure success rate was 70.48%, with no significant difference between groups (72.38% vs. 68.18%; log-rank test, *p* = 0.27, Figure 4).

In multi-variable Cox regression analysis, lower LVEF (OR = 0.947, *p* = 0.001), additional AT ablation (OR = 1.996, *p* = 0.002) and spontaneous non-PV trigger (OR = 1.873, *p* = 0.033) were independent predictors for recurrence (Supplemental Table 3).

### Discussion

In this study, we examined the prevalence, predictors, and outcomes of additional ablations beyond PVI in patients with PAF for the index procedure. Our result revealed that up to 44.88% of patients underwent additional ablation, which was predicted by lower LVEF, AF episode before the procedure, AF episode during the procedure, and AF episode induced after PVI. After a five-year follow-up, 70% of patients were free from AT/AF recurrence.

### Ablation Beyond PVI

Since PVI was identified as the basis of AF ablation, the pursuit for better ablation outcome has never stopped (1, 2). In PAF patients, non-PV trigger ablation is commonly performed (17, 18), while some other methods of ablation beyond PVI have also been tried. Routine linear, complex fractionated electrograms, and rotor ablations were reported associated with improved clinical outcomes compared with PVI alone (6, 7, 9, 19). Faustino et al. reported a stepwise ablation strategy that accomplished a 90.7% success rate after 12-month follow-up in PAF patients (20). However, in a more recent study, elimination of triggers as an end point of ablation in patients with PAF showed a lower recurrence compared with stepwise substrate modifications (21). A meta-analysis, which included 145 studies with 23 263 patients, revealed that PVI plus studies were associated with improved outcomes, while the large residual heterogeneity lowered its confidence level (22). These ambiguous results hindered the wide clinical practice of ablation beyond PVI in PAF patients. Moreover, non-contiguous ablation lesions performed may further increase the incidence of iatrogenic arrhythmia (23). Thus, in our center, the lesions were delivered based on objective evidences, including clinical recordings, provocation results, and voltage mapping, which was consistent with previous studies (16). Accordingly, ablations beyond PVI were mainly focused on concomitant arrhythmia, non-PV trigger, and substrate modifications. No complex fractionated atrial electrogram or routine linear ablation was performed.

### Characteristics of Additional Ablation

In our study, additional ablation beyond PVI was frequently performed mainly because of a very high incidence of concomitant arrhythmia, where AFL accounted for the majority. The coexistence of AF and AFL is frequently observed in clinical practice, and their relationship has been well recognized (12). The incidence of AT and atrioventricular node reentrant tachycardia were consistent with previous findings (11, 24), and corresponding successive ablations were associated with improved outcomes. Non-PV trigger ablations in our study accounted for 15.05% of additional ablations involving 52 patients. The reported prevalence and distribution of non-PV triggers varied in different studies, which may be due to different populations, definitions, and provocation protocols (10). Successful detection and elimination of non-PV triggers in PAF patients has been shown associated with better outcomes (17).

In a previous study (25), we used high-density mapping of the LA during sinus rhythm in different AF populations and found that as AF progressed, patients exhibited more low voltage zones, longer LA conduction times, and more complex electrograms. According to Rolf's finding, LVZs can be found in 10% of patients with PAF (26). In our study, considering the internal relationship between atrial fibrosis, LVZ, and AF, substrate modification was accordingly performed in 47 (8.75%) patients, which was comparable to Rolf's work.

### Predictors of Additional Ablation

Predictors of additional ablations in PAF patients have not been well illustrated in previous studies. Zhao et al. (27) reported a correlation between low-voltage and the presence of non-PV triggers in PAF patients. In the study by Piorkowski et al. age, sex, AF type, and left atrial appendage velocity were independently associated with LVZs (26). As far as we know, our work was the first study that describe all additional ablations, including concomitant arrhythmia, non-PV trigger, and LVZ ablations in one study. In our study, lower LVEF, AF episode before the procedure, AF episode during the procedure, and AF episode induced after PVI resulted as independent predictors for additional ablation. Risk stratification for the needs of additional ablation using these clinical parameters may support perioperative preparation. And, after PVI, special attention should be paid to patients with these risk factors.

### The Outcome of Additional Ablation

Several studies had presented 5–6 years of follow-up data after PVI in PAF patients with a success rate ranging from 46 to 56% (3–5). In our study, a 5-years follow-up revealed a free rate of 70%. With reference to baseline characteristics, patients who underwent additional ablations presented a larger left atrium and lower LVEF, which were previously reported as the risk factors of recurrence (18, 28–30). Moreover, non-PV triggers and worse left atrium substrate may further worsen the outcome (13, 31). Though presenting a relative worse clinic characteristic, patients underwent additional ablation still showed a 68.18% success rate after long follow-up. According to Piorkowski's findings (26), limited substrate modification of LVZs may potentially have a compensatory effect for the impaired outcome in patients with endocardial structural defects. In our study, this effect might be expended with additional ablation in patients with worse baseline conditions. Nonetheless, further prospective, controlled clinical trials containing more variables, such as obstructive sleep apnea and PV anatomy (32), are needed to further clarify these results.

## Limitations

This study has some limitations needed to point out, such as a retrospective design. However, the clinical characteristics, procedure-related data, and follow-up were prospectively collected, and the population was relatively large, helping to minimize bias. Second, although all patients were educated about the follow-up, 17% of patients were lost in 5 years. However, the loss was comparable in both groups. Third, 24-h Holter and telephone interviews, rather than an insert able cardiac monitor, have the potential to underestimate recurrence. Last but not the least, there was no control group of patients undergoing PVI only which would strengthen our findings. Further prospective randomized study would better clarify this hypothesis.

## Conclusions

Additional ablations were common in patients with PAF for index procedure. Lower LVEF and AF episodes before, during the procedure, and induced after PVI may predicts additional ablation.

## Data Availability Statement

The raw data supporting the conclusions of this article will be made available by the authors, without undue reservation.

## Ethics Statement

The studies involving human participants were reviewed and approved by The Institutional Review Board of Nanjing Medical University. The patients/participants provided their written informed consent to participate in this study.

## Author Contributions

All authors listed have made a substantial, direct and intellectual contribution to the work, and approved it for publication.

## Conflict of Interest

The authors declare that the research was conducted in the absence of any commercial or financial relationships that could be construed as a potential conflict of interest.
