# Supplementary material for: Prevalence and Predictors of Additional Ablation Beyond Pulmonary Vein Isolation in Patients With Paroxysmal Atrial Fibrillation
Source: Front Cardiovasc Med. 2021 Jul 20;8:690297. doi: 10.3389/fcvm.2021.690297 (PMC8329378; doi:10.3389/fcvm.2021.690297)
Supplement: Supplementary file 1 [file Data_Sheet_1.docx]

**SUPPLEMENTAL MATERIAL**

**Article title:**

Prevalence and Predictors of Additional Ablation beyond Pulmonary Vein Isolation in Patients with Paroxysmal Atrial Fibrillation

**Journal name:**

Frontiers in Cardiovascular Medicine

**Author names:**

Xin Xie^1^, MD; Gang Yang^2^, MD; Xiaorong Li^1^, PhD; Jinbo Yu^1^, MD; Fengxiang Zhang^2^, MD; Weizhu Ju^2^, MD; Hongwu Chen^2^, MD; Mingfang Li^2^, MD; Kai Gu^2^, MD; Dian Cheng^1^, MD; Xuecheng Wang^1^, MD; Yizhang Wu^1^, MD; Jian Zhou^1^, MD; Xiaoqian Zhou^1^, MD; Baowei Zhang^1^, MD; Pipin Kojodjojo^3^, PhD; Kejiang Cao^2^, MD; Bing Yang^1^, MD, FHRS; Minglong Chen^2^, MD, FHRS

**Affiliation:**

1.Department of Cardiology, Shanghai East Hospital, Tongji University School of Medicine

2.Department of Cardiology, The First Affiliated Hospital of Nanjing Medical University

3. Division of Cardiology, Ng Teng Fong General Hospital, Singapore

**E-mail address of the corresponding author:**

E-mail: ybheart@163.com

**Supplemental Table S1.** The Distribution of Non-PV Triggers Location

| Origin of Non-PV triggers | Spontaneous Onset  N | Provocation with Iso  N | Programmed stimulation  N | Provocation with ATP  N | Subjective  Ablation  N | N |
| --- | --- | --- | --- | --- | --- | --- |
| Superior vena cava | 27 | 6 | 1 | 7 | 4 | 45(80.36%) |
| Fossa ovalis | 1 | 2 | 0 | 0 | 0 | 3(5.36%) |
| Coronary sinus | 2 | 0 | 0 | 1 | 0 | 3(5.36%) |
| Crista terminalis | 1 | 1 | 0 | 0 | 0 | 2(3.57%) |
| Roof of left atrium | 0 | 1 | 0 | 0 | 0 | 1(1.79%) |
| Left superior vena cava | 1 | 0 | 0 | 0 | 0 | 1(1.79%) |
| Total | 33(58.93%) | 10(17.86%) | 1(1.79%) | 8(14.29%) | 4(7.14%) | 56(100%) |

ATP: adenosine triphosphate; Iso: isoprenaline; PV: pulmonary vein.

**Supplemental Table S2.** Uni- and Multi-Variate Logistic Regression Analyses for Potential Factors Associated with Additional Ablation.

| Characteristics | Univariate analyses | |  | Multivariate analyses | |
| --- | --- | --- | --- | --- | --- |
|  | OR (95% CI) | *P* Value |  | OR (95% CI) | *P* Value |
| Age | 1.008 (0.992-1.025) | 0.339 |  |  |  |
| Male | 0.772 (0.544-1.093) | 0.145 |  |  |  |
| History of PAF | 1.001 (0.998-1.005) | 0.357 |  |  |  |
| Smoke | 0.779 (0.489-1.241) | 0.293 |  |  |  |
| Alcohol consumption | 1.009 (0.611-1.667) | 0.972 |  |  |  |
| CAD | 0.732 (0.402-1.331) | 0.306 |  |  |  |
| Hypertension | 0.846 (0.601-1.189) | 0.335 |  |  |  |
| DM | 1.309 (0.756-2.269) | 0.337 |  |  |  |
| CHA2DS2-VASc |  |  |  |  |  |
| 0 | Ref |  |  |  |  |
| 1 | 0.659 (0.431-1.008) | 0.055 |  |  |  |
| 2 | 1.098 (0.677-1.781) | 0.704 |  |  |  |
| ≥3 | 1.443 (0.851-2.449) | 0.174 |  |  |  |
| LAD | 1.045 (1.008-1.083) | 0.018 |  |  |  |
| LVEF | 0.933 (0.885-0.984) | 0.010 |  | 0.937 (0.889-0.987) | 0.015 |
| AF episodes before procedure | 2.977 (1.630-5.435) | <0.001 |  | 2.990 (1.586-5.637) | 0.001 |
| AF episodes during procedure | 1.708 (1.120-2.606) | 0.013 |  | 1.998 (1.280-3.118) | 0.002 |
| AF episode Needed DCCV after PVI | 3.576 (1.841-6.944) | <0.001 |  |  |  |
| AF episode induced after PVI | 16.258 (3.802-69.527) | <0.001 |  | 15.958 (3.677-69.255) | <0.001 |

CAD: Coronary artery disease; DCCV: direct current cardioversion; DM: Diabetes mellitus; LAD: diameter of left atrium; LVEF: left ventricular ejection fraction; PAF: paroxysmal atrial fibrillation; PVI: pulmonary vein isolation.

**Supplemental Table S3.** Uni- and Multi- Variate Analyses of Predictors for Recurrence

| Characteristics | Univariate analyses | |  | Multivariate analyses | |
| --- | --- | --- | --- | --- | --- |
|  | HR (95% CI) | *P* Value |  | HR (95% CI) | *P* Value |
| Age | 1.005(0.988-1.022) | 0.580 |  |  |  |
| Male | 0.978(0.686-1.394) | 0.903 |  |  |  |
| History of PAF | 1.002(1.000-1.005) | 0.045 |  |  |  |
| Smoke | 1.186(0.761-1.848) | 0.451 |  |  |  |
| Alcohol consumption | 1.247(0,774-2.009) | 0.365 |  |  |  |
| CAD | 0.935(0.504-1.735) | 0.832 |  |  |  |
| CHF | 3.291(0.813-13.316) | 0.095 |  |  |  |
| Hypertension | 0.942(0.666-1.332) | 0.735 |  |  |  |
| DM | 1.286(0.762-2.171) | 0.347 |  |  |  |
| CHA2DS2-VASc |  |  |  |  |  |
| 0 | Ref |  |  |  |  |
| 1 | 0.766(0.489-1.199) | 0.244 |  |  |  |
| 2 | 1.129(0.707-1.803) | 0.612 |  |  |  |
| ≥3 | 1.130(0.679-1.882) | 0.637 |  |  |  |
| LAD | 1.028(0.992-1.066) | 0.126 |  |  |  |
| LVEF | 0.948(0.920-0.977) | <0.001 |  | 0.947(0.918-0.977) | 0.001 |
| AF episodes before procedure | 1.784(1.096-2.905) | 0.020 |  |  |  |
| AF episodes during procedure | 1.058(0.696-1.609) | 0.791 |  |  |  |
| AF episodes need DCCV | 1.500(0.875-2.570) | 0.140 |  |  |  |
| AF episode induced after PVI | 0.608(0.225-1.646) | 0.328 |  |  |  |
| Additional ablation | 1.216(0.861-1.717) | 0.267 |  |  |  |
| Concomitant arrhythmia | 0.981(0.685-1.404) | 0.914 |  |  |  |
| Additional AFL ablation | 0.767(0.504-1.165) | 0.213 |  |  |  |
| Additional AT ablation | 2.045(1.312-3.187) | 0.002 |  | 1.996(1.277-3.122) | 0.002 |
| Additional SVT ablation | 0.848(0.396-1.818) | 0.673 |  |  |  |
| Additional PAC ablation | 1.086(0.401-2.939) | 0.871 |  |  |  |
| Inducible AFL | 0.388(0.054-2.777) | 0.346 |  |  |  |
| Inducible AT | 1.981(0.968-4.052) | 0.061 |  |  |  |
| Inducible SVT | 0.962(0.355-2.604) | 0.939 |  |  |  |
| Inducible PAC | 0.049(0-1878) | 0.576 |  |  |  |
| Non-PV trigger | 1.540(0.925-2.566) | 0.097 |  |  |  |
| Spontaneous Non-PV trigger | 2.034(1.146-3.611) | 0.015 |  | 1.873(1.051-3.336) | 0.033 |
| Inducible Non-PV trigger | 0.947(0.350-2.563) | 0.914 |  |  |  |
| Substrate modification | 1.821(1.078-3.074) | 0.025 |  |  |  |

AFL: typical atrial flutter; AT: atrial tachycardia; CAD: Coronary artery disease; CHF: Congestive heart failure; DM: Diabetes mellitus; DCCV: direct current cardioversion; LAD: diameter of left atrium; LVEF: left ventricular ejection fraction; PAC: premature atrial contraction; PAF: paroxysmal atrial fibrillation; PV: pulmonary vein; PVI: pulmonary vein isolation; SVT: supraventricular tachycardia.
